# Prevalence and predictors of HCV infection among hospitalized psychiatric patients in Poland

**DOI:** 10.3389/fpsyt.2026.1835310

**Published:** 2026-07-13

**Authors:** Krzysztof Tomasiewicz, Piotr Gałecki, Jerzy Jaroszewicz, Jolanta Opoka, Małgorzata Pawłowska, Anna Piekarska, Krzysztof Simon, Dorota Zarębska-Michaluk, Magdalena Władysiuk, Tomasz Prycel, Sławomir Ros, Dominik Obierzyński, Waldemar Halota, Robert Flisiak

**Affiliations:** 1 Department of Infectious Diseases, Medical University of Lublin, Lublin, Poland; 2 Department of Adult Psychiatry, Medical University of Lodz, Lódź, Poland; 3 Department of Infectious Diseases and Hepatology, Medical University of Silesia, Katowice, Poland; 4 Department of Infectious Diseases, Medical University of Warsaw, Warsaw, Poland; 5 Department of Infectious Diseases and Hepatology, Faculty of Medicine, Nicolaus Copernicus University, Toruń, Poland; 6 Department of Infectious Diseases and Hepatology, Medical University of Lodz, Lódź, Poland; 7 Department of Infectious Diseases and Hepatology, Wroclaw Medical University, Wrocław, Poland; 8 Department of Infectious Diseases and Allergology, Jan Kochanowski University, Kielce, Poland; 9 Collegium Medicum, Jagiellonian University, Kraków, Poland; 10 Central and Eastern European Society of Technology Assessment in Health Care, Kraków, Poland; 11 Gilead Sciences Poland, Warsaw, Poland; 12 Department of Infectious Diseases and Hepatology, Medical University of Bialystok, Białystok, Poland

**Keywords:** HCV infection, mental disorders, Poland, psychiatric patients, risk factors

## Abstract

**Background:**

Patients with mental disorders are at increased risk of hepatitis C virus (HCV) infection, yet the prevalence of HCV among patients in Polish psychiatric ward remains unknown.

**Methods:**

A cross-sectional survey was conducted in Poland from 2021 to 2023 in psychiatric wards. Adults recently hospitalized with at least one current mental health condition were eligible and 12–897 individuals were tested for anti HCV antibodies. Patients with confirmed active HCV infection (subsequent nucleic acid testing) were offered treatment.

**Results:**

Of the 12,897 individuals who constituted the sample, 64% were male, and the median age was 45 years (interquartile range: 35–58 years). Among those subjects, 226 patients (representing 1.8% of the total) exhibited a positive HCV antibody test result, and 172 of these patients had active HCV infection (the prevalence of 1.3%). Multivariate analysis identified five risk factors for HCV infection within the investigated population: intravenous drug use (odds ratio [OR] = 9.40; 95% confidence interval [CI]: 6.12–14.44; p<0.001), diagnosed and treated liver disease (OR = 6.17; 95% CI: 4.10–9.26; p < 0.001); blood transfusions prior to 1992 (OR = 2.61; 95% CI: 1.29–5.28; p=0.008), diagnosis of mental and behavioral disorders due to psychoactive substance abuse (OR = 1.97; 95% CI: 1.34–2.91; p=0.001) and previous incarceration (OR = 1.62; 95% CI: 1.08–2.43, p=0.018).

**Conclusions:**

High prevalence of HCV infection in patients with mental health disorders and specifically in patients who used intravenous drugs suggests the importance of screening for HCV in this vulnerable population.

## 1 Introduction

Hepatitis C virus (HCV) is a major public health concern with a global prevalence that poses a serious risk to liver health, potentially leading to cirrhosis and hepatocellular carcinoma (1). Despite the availability of effective antiviral treatments that can cure the infection, HCV remains challenging due to a large number of undiagnosed cases. This issue is particularly relevant for populations with increased HCV prevalence or limited access to screening, such as patients hospitalized for mental disorders. These individuals are more likely to engage in behaviors that increase the risk of both for communicable and non-communicable diseases (2).

Patients with psychiatric disorders are a vulnerable group with several risk factors for HCV and other blood borne infections (3). These include a high prevalence of intravenous drug use, homelessness, incarceration, and limited access to health care, all of which elevate the risk of HCV acquisition and transmission. Additionally, the psychiatric symptoms like reduced awareness, impaired judgment and possible less care for somatic health, may contribute to risky behaviors that facilitate HCV transmission (4).

The epidemiology of HCV among patients with mental disorders seems to by insufficiently characterized, partly due to the lack of routine HCV screening in psychiatric settings. Contributing factors include the separation of mental and general health services, stigma associated with mental health disorders, which may result in treating patients with mental disorder less seriously than those of the general population, and the lack of specific guidelines recommending HCV screening in psychiatric patients (5–7).

Historically, physical and psychiatric healthcare have been divided, with necessary preventive measures, such as screening and vaccination, often neglected in psychiatric settings. The lack of routine HCV screening in psychiatric units reflects a gap in the comprehensive care model, which ideally should address both mental and physical health (6, 8).

The importance of HCV screening in psychiatric settings cannot be overstated, as the early detection and treatment of HCV can prevent severe liver disease and reduce the overall disease burden of HCV. Timely diagnosis may also reduce the risk of extrahepatic conditions related to this infection, including cryoglobulinemia, renal disease, dermatologic disorders, diabetes, lymphomas and, last but not least, psychiatric diseases (9). From a public health perspective, identifying and treating HCV in psychiatric patients can play a pivotal role in controlling the spread of the virus, especially in closed environments like psychiatric hospitals where transmission risk is higher.

In 2016, the World Health Organization (WHO) launched the Global Health Sector Strategy on Hepatitis, aiming to eliminate HCV by 2030. This strategy requires countries to diagnose 90% of all HCV cases and to treat 80% of those diagnosed (10). It is believed that this approach will result in reducing the number of deaths from viral hepatitis C. A significant advancement supporting these objectives is the development and increasing availability of highly effective, all-oral, interferon-free direct-acting antivirals (DAAs). These medications facilitate the cure of HCV in the majority of individuals who access healthcare services and adhere to the prescribed treatment regimen (11). According to Polish and European Recommendations HCV-infected individuals should begin treatment without delay. The antiviral medications employed in the management of HCV infection reduce the risk of developing cirrhosis and hepatocellular carcinoma and prevent new infections, making HCV a engageable and preventable health concern (12, 13).

This article explores the rationale for routine HCV screening among hospitalized psychiatric patients, examining the epidemiological overlap between psychiatric disorders and HCV infection, barriers to screening in psychiatric settings, and the potential benefits of integrating HCV screening into routine psychiatric care. It also discusses the broader implications of such integration, including improved patient outcomes, reduced healthcare costs, and contribution to global HCV elimination effort.

## 2 Methods

The Polish HCV initiative was a comprehensive cross-sectional research study conducted in Poland from 2021 to 2023, focusing on individuals hospitalized for mental health in 24 facilities. It is estimated that the study population covered over 7% of patients hospitalized due to the mental diseases. The main objective of this initiative was to conduct HCV screening and collect data on prevalence and risk factors associated with HCV infection, including drug dependence. All participants received education on the prevention, transmission, diagnosis, and treatment of liver diseases, with a specific focus on hepatitis C. This educational program was initially targeted at hospital staff and subsequently extended to patients through sessions led by educators or trained nurses. Recruitment in the study was conducted in both in both inpatient and emergency departments. Eligible participants were Polish citizens, aged 18 or older, who had been recently hospitalized and diagnosed with at least one current mental health condition. All individuals involved in the study provided written consent to participate. Diagnosis of hepatitis C Virus infection was confirmed through positive HCV antibody screening and subsequent nucleic acid testing (NAT). This publication reports on data collected from this representative cohort of hospitalized patients with mental health disorders, selected based on the practical and comprehensive design of the study. Participants were interviewed by healthcare professionals, who administered a detailed questionnaire to collect the necessary data. All patients diagnosed with HCV infection were offered on-site treatment or were referred to the outpatient clinic.

### 2.1 Study design and participants

#### 2.1.1 Statistical analysis

The analysis examined the relationship between confirmed HCV infection, as determined by genetic testing, and various population characteristics. Logistic regression was chosen as the method for this statistical investigation. First, univariate logistic regression was performed on the variables of interest. Subsequently, only those variables that significantly influenced the occurrence of HCV infection in the univariate analysis were included in a more complex multivariate analysis. Throughout these analyses, a p-value of less than 0.05 was considered to indicate statistical significance.

IN addition, the characteristics of the entire study population were described in detail, including the subdivisions between healthy individuals (those without an HCV infection) and those who were HCV-positive. The statistical significance of differences between these groups for each characteristic was assessed using the Chi-square test, Mann-Whitney U test, and Fisher’s exact test as appropriate. This comparative analysis allowed for the evaluation of factors not included in the logistic regression, such as categorical or non-ordinal variables, such as employment status, or variables with substantial missing data, such as tests for HBV (HBsAg) and HIV (human immunodeficiency virus).

The statistical computations were carried out using the Real Statistics Data Analysis Tool add-on in MS Excel. In the regression analyses performed, data entries from participants who did not provide information on their history of incarceration or intravenous drug use were excluded from the study.

## 3 Results

Among the 12,897 individuals tested, 226 patients, i.e. 1.8% of study population, had a positive HCV antibody test result. For 221 of these patients, the HCV RNA test result was known, indicating that 172 of them had active HCV infection. The prevalence of active HCV infection was then estimated to be 1.3%. The baseline characteristics of the population in general and according to HCV RNA status are presented in **Table 1**.

**Table 1:** Characteristics of patients according to the result of HCV RNA test – all patients.

Characteristics		Total	Negative HCV antibody or HCV RNA test	Positive result of the HCV RNA test	P value
Patients, No. (%)		12 897 (100%)	12 725 (100%)	172 (100%)	–
Age: mean (SD) median [IQR] range		46.73 (15.80) 45 [35; 58] 18–102	46.74 (15.86) 45 [35; 58] 18–102	45.91 (10.49) 43 [40; 51] 22–75	0.934
Sex, No. (%)	Man	8248 (64%)	8111 (64%)	132 (77%)	**<0.001**
	Woman	4649 (36%)	4614 (36%)	40 (23%)	
Education, No. (%)	Basic. or incomplete	2852 (22%)	2810 (22%)	42 (24%)	0.098
	Basic vocational training	3785 (29%)	3727 (29%)	58 (34%)	
	Secondary or post-secondary	4699 (36%)	4638 (36%)	61 (35%)	
	Higher	1561 (12%)	1550 (12%)	11 (6%)	
Place of residence, No. (%)	Village	4052 (31%)	4021 (32%)	31 (18%)	**<0.001**
	Town up to 20,000 people	2185 (17%)	2156 (17%)	29 (17%)	
	Town up to 100,000 people	2538 (20%)	2501 (20%)	37 (22%)	
	City up to 500,000 people	1809 (14%)	1782 (14%)	27 (16%)	
	City of over 500,000 people	2313 (18%)	2265 (18%)	48 (28%)	
Alcohol consumption, No. (%)	Abstinent	3627 (28%)	3585 (28%)	42 (24%)	0.221
	Occasional consumption of alcohol	3394 (26%)	3348 (26%)	46 (27%)	
	Moderate consumption of alcohol	1833 (14%)	1814 (14%)	19 (11%)	
	High risk group	4043 (31%)	3978 (31%)	65 (38%)	
Professional activity, No. (%)	Manual worker (excluding agriculture)	3459 (27%)	3400 (27%)	59 (34%)	**0.001**
	White-collar worker	1325 (10%)	1316 (10%)	9 (5%)	
	Farmer	296 (2%)	295 (2%)	1 (1%)	
	Unemployed, looking for work	2251 (17%)	2209 (17%)	42 (24%)	
	Unemployed, not looking for a job (e.g. housewife)	796 (6%)	782 (6%)	14 (8%)	
	Retired	1676 (13%)	1666 (13%)	10 (6%)	
	Pensioner	2714 (21%)	2679 (21%)	35 (20%)	
	Pupil or student	380 (3%)	378 (3%)	2 (1%)	
Disability pension, No. (%)	Yes	2977 (23%)	2937 (23%)	40 (23%)	0.957
	No	9920 (77%)	9788 (77%)	132 (77%)	
Homelessness, No. (%)	Yes	716 (6%)	691 (5%)	25 (15%)	**<0.001**
	No	12181 (94%)	12034 (95%)	147 (85%)	
Diagnosed and treated liver disease, No. (%)	Yes	663 (5%)	597 (5%)	66 (38%)	**<0.001**
	No	12232 (95%)	12127 (95%)	105 (61%)	
Any previous treatment for hepatitis C, No. (%)	Yes	167 (1%)	138 (1%)	29 (17%)	**<0.001**
	No	12728 (99%)	12586 (99%)	142 (83%)	
Any test for HCV in the past, No. (%)	Yes	1331 (10%)	1238 (10%)	93 (54%)	**<0.001**
	No	11564 (90%)	11486 (90%)	78 (45%)	
Elevated levels of transaminases (ALT and/or AST), No. (%)	Yes	2204 (17%)	2142 (17%)	62 (36%)	**<0.001**
	No	6967 (54%)	6909 (54%)	58 (34%)	
	No data	3725 (29%)	3673 (29%)	52 (30%)	
Number of surgical procedures: mean (SD) median [IQR] range		1.11 (1.95) 1 [0; 2] 0–70	1.11 (1.95) 1 [0; 2] 0–70	1.45 (1.98) 1 [0; 2] 0–15	**0.003**
Number of hospital stays throughout life: mean (9SD) median [IQR] range		7.03 (8.40) 5 [3;8] 0–170	6.96 (8.15) 5 [3;8] 0–170	11.83 (18.76) 7 [4; 15] 0–170	**<0.001**
Blood transfusions before 1992, No. (%)	Yes	317 (2%)	302 (2%)	15 (9%)	**<0.001**
	No	12579 (98%)	12423 (98%)	156 (91%)	
Work involving exposure to blood, No. (%)	Yes	358 (3%)	351 (3%)	7 (4%)	0.291
	No	12538 (97%)	12374 (97%)	164 (95%)	
Minor cosmetic procedures in medical history, No. (%)	Yes	1655 (13%)	1632 (13%)	23 (13%)	0.808
	No	11241 (87%)	11093 (87%)	148 (86%)	
Tatoo, No. (%)	Yes	3505 (27%)	3425 (27%)	80 (47%)	**<0.001**
	No	9391 (73%)	9300 (73%)	91 (53%)	
Stays in a penal institution (e.g., prison, detention center), No. (%)	Yes	2362 (18%)	2283 (18%)	79 (46%)	**<0.001**
	No	10092 (78%)	10008 (79%)	84 (49%)	
	No response	443 (3%)	434 (3%)	9 (5%)	
Self-harm, No. (%)	Yes	2540 (20%)	2480 (19%)	60 (35%)	**<0.001**
	No	10356 (80%)	10245 (81%)	111 (65%)	
The use of intravenous drugs at any point in time, No. (%)	Yes	780 (6%)	689 (5%)	91 (53%)	**<0.001**
	No	11870 (92%)	11794 (93%)	76 (44%)	
	No response	247 (2%)	242 (2%)	5 (3%)	
The use of any drugs other than intravenously at any point of time, No. (%)	Yes	3833 (30%)	3723 (29%)	110 (64%)	**<0.001**
	No	8759 (68%)	8702 (68%)	57 (33%)	
	No response	305 (2%)	300 (2%)	5 (3%)	
Recordered diagnosis of mental and behavioural disorders due to psychoactive substance abuse (ICD-10 F10-F19), No. (%)	Yes	5031 (39%)	4913 (39%)	118 (69%)	**<0.001**
	No	7866 (61%)	7812 (61%)	54 (31%)	
Ever having undergone dialysis treatment, No. (%)	Yes	46 (0%)	45 (0%)	1 (1%)	0.614
	No	12850 (100%)	12680 (100%)	170 (99%)	
HBsAg test, No. (%)	None (not performed/unknown)	11220 (87%)	11097 (87%)	123 (72%)	**<0.001**
	negative	1614 (13%)	1578 (12%)	36 (21%)	
	positive	63 (0%)	50 (0%)	13 (8%)	
HIV test, No. (%)	None (not performed/unknown)	10646 (83%)	10547 (83%)	99 (58%)	**<0.001**
	negative	2193 (17%)	2133 (17%)	60 (35%)	
	positive	57 (0%)	45 (0%)	12 (7%)	

P values in bold are statistically significant (P < 0.05).

Univariate analysis identified a number of variables that significantly influenced HCV infection (**Table 2**).

**Table 2:** Predictive factors of positive HCV RNA test - all patients.

Variable	Univatiate analysis				Multivariate analysis			
	OR	Confidence interval		P value	OR	Confidence interval		P value
Age	0.995	0.985	1.005	0.291	–	–	–	–
Sex (woman=0, man=1)	2.075	1.424	3.025	**<0.001**	1.099	0.714	1.691	0.669
Education (basic = 1, basic vocational training = 2, secondary or post-secondary = 3, higher = 4)	0.847	0.719	0.998	**0.047**	0.978	0.807	1.186	0.824
Place of residence (village = 1; town up to 20k=2; town up to 100k; city up to 500k; city over 500k)	1.262	1.136	1.402	**<0.001**	1.023	0.903	1.159	0.716
Alcohol consumption (abstinent=1; occasional=2; moderate =3, high risk=4)	1.108	0.973	1.263	0.122	–	–	–	–
Homelessness (no=0, yes=1)	2.951	1.884	4.622	**<0.001**	1.412	0.835	2.386	0.198
Diagnosed and treated liver disease (no=0, yes=1)	13.260	9.555	18.401	**<0.001**	6.165	4.104	9.260	**<0.001**
Previous treatment for hepatitis C (no=0, yes= 1)	17.355	11.031	27.306	**<0.001**	1.327	0.722	2.438	0.363
Number of surgical procedures	1.004	0.990	1.019	0.614	–	–	–	–
Number of hospital stays throughout life	1.029	1.020	1.038	**<0.001**	1.010	0.999	1.022	0.084
Blood transfusions before 1992 (no=0, yes=1)	3.261	1.790	5.940	**<0.001**	2.608	1.289	5.278	**0.008**
Work involving exposure to blood (no=0, yes=1)	1.384	0.608	3.151	0.439	–	–	–	–
Minor cosmetic procedures in medical history (no=0, yes=1)	0.871	0.531	1.426	0.582	–	–	–	–
Tatoo (no=0, yes=1)	2.665	1.948	3.646	**<0.001**	0.927	0.626	1.374	0.706
History of imprisonment (no=0, yes=1)	4.229	3.088	5.790	**<0.001**	1.623	1.085	2.429	**0.018**
Self-harm (no=0, yes=1)	2.320	1.672	3.218	**<0.001**	0.738	0.492	1.107	0.142
Ever using intravenous drugs (no=0, yes=1)	20.789	15.069	28.679	**<0.001**	9.398	6.116	14.441	**<0.001**
Ever using drugs in a form other than intravenous (no=0, yes=1)	4.635	3.330	6.450	**<0.001**	1.373	0.885	2.130	0.157
Recordered diagnosis of mental and behavioural disorders due to psychoactive substance abuse (ICD-10 F10-F19) (no=0, yes=1)	4.010	2.83	5.67	**<0.001**	1.974	1.340	2.910	**0.001**
Dialysis therapy now or in the past (no=0, yes=1)	1.865	0.255	13.640	0.539	–	–	–	–

P values in bold are statistically significant (P < 0.05).

The largest discrepancies between the groups were observed with regard to past intravenous drug use, the presence of liver disease and previous treatment for hepatitis C. The multivariate analysis conducted as part of the study identified a number of key variables that were found to significantly influence the occurrence of HCV infection among the entire patient population under investigation. Each of these factors was found to carry its own degree of risk, as quantified by their respective odds ratios (ORs).:

- The analysis identified intravenous drug use as the primary factor contributing to a positive HCV RNA test, with an odds ratio of 9.40 (95% CI: 6.12–14.44; p<0.001). This emphasizes the considerable risk associated with intravenous drug use, thereby underscoring the vital necessity for targeted interventions and educational initiatives within populations prone to this behavior.
- The presence of diagnosed and treated liver disease was also identified as a significant risk factor, with an odds ratio of 6.17 (95% CI: 4.10–9.26; p < 0.001). This finding indicates that individuals with a history of liver disease, potentially exacerbated by or contributing to HCV infection, are at a markedly elevated risk and necessitate meticulous monitoring and management.
- The odds ratio for blood transfusions prior to 1992 was 2.61 (95% CI: 1.29–5.28; p=0.008). Prior to the implementation of more rigorous screening protocols in the early 1990s, blood transfusions carried a heightened risk of HCV transmission, underscoring the significance of historical medical interventions as a contributing factor to the current prevalence of HCV.
- A recorded diagnosis of mental and behavioral disorders due to psychoactive substance abuse (ICD-10 F10-F19) had an OR of 1.97 (95% CI: 1.34–2.91; p=0.001). This finding indicates that the most frequently reported group in the analyzed population is also the group with the substantially increased high-risk of HCV.
- Those who had ever been in prison exhibited a diminished yet still markedly elevated risk of HCV infection, with an OR of 1.62 (95% CI: 1.08–2.43, p=0.018). This finding reflects the heightened risk of HCV infection associated with incarceration, potentially attributable to factors such as elevated rates of risky behaviors.

These findings underscore the intricate nature of HCV transmission and the multifactorial character of risk in the studied population. It is therefore evident that effective prevention and intervention strategies must address these diverse risk factors through comprehensive approaches that include robust screening programs, targeted education for high-risk groups, and interventions designed to reduce harm and promote health in settings such as prisons and drug treatment facilities.

A review of the baseline characteristics of patients with confirmed HCV infection revealed that in only 19 out of 172 cases did the patients confirm the absence of any of the five factors, which demonstrated their association with a positive HCV RNA test in a multivariate regression analysis.

**Table 3** summarizes the prevalence of confirmed HCV infection across major diagnostic groups in ICD-10 among individuals with mental disorders. Consistent with the logistic regression results, mental and behavioral disorders due to psychoactive substance use (F10–F19) clearly stand out, with a markedly higher proportion of HCV-positive cases than any other category.

**Table 3:** Prevalence of active HCV infection across ICD-10 diagnostic groups.

ICD-10 group	n	N	Percentage of patients with diagnosed HCV (95%CI)
F00-F09 Organic, including symptomatic, mental disorders	6	1116	0.54% (0.20–1.17%)
F10-F19 Mental and behavioural disorders due to psychoactive substance use	118	5033	2.34% (1.94%–2.08%)
F20-F29 Schizophrenia, schizotypal and delusional disorders	33	3879	0.85% (0.59%–1.19%)
F30-F48 Mood, neurotic, stress-related and somatoform disorders	13	2083	0.62% (0.33%–1.06%)
F50-F69 Behavioural syndromes and personality disorders	0	339	0.00% (0.00%–1.08%)
F70-F98 - Neurodevelopmental and childhood behavioural disorders	1	293	0.34% (0.01%–1.89%)
Others/unidentified	3	401	0.75% (0.15%–2.17%)

Some patients have more than 1 diagnosis.

Outside this group, HCV infection appears infrequent and unevenly distributed. Schizophrenia spectrum disorders (F20–F29) show a modestly elevated prevalence compared with other non–substance-related categories. By contrast, the remaining groups – including organic, mood and neurotic, personality-related, and neurodevelopmental disorders – show only sporadic or no cases, underscoring the strong concentration of HCV infection within substance-related conditions.

## 4 Discussion

The implementation of HCV screening and treatment strategies varies worldwide. Some countries have adopted universal one-time HCV screening; while others focus on high-risk populations. In 2019, the Swiss Federal Office of Public Health prioritized screening only among high-risk individuals instead of an general screening programme (14). In contrast, Lithuania launched an universal screening program in May 2022. It involved anti-HCV testing by general practitioners in two options: (1) one-time testing for individuals born between 1945 and 1994 and (2) annual HCV testing for persons who inject drugs or are living with HIV regardless of age. Between 5 May 2022 and 31 October 2023, 1.03 million Lithuanians were tested, including nearly 60% of those born between 1945 and 1994 (15, 16).

Systematic screening, whether universal or targeted, is essential for identifying cases achieving global elimination goals set by WHO (2). Our study provides evidence supporting the expansion of HCV screening and treatment initiatives aimed at virus elimination. Early administration of direct-acting antivirals (DAAs) after diagnosis may markedly reduce disease progression in high-prevalence groups, including individuals with mental disorders, incarcerated persons, and people who inject drugs. This approach is not only vital for the management of individual health outcomes, but also for broader public health impact. A recent Polish study confirmed that direct-acting antiviral treatment is highly effective in hepatitis C virus patients with mental disorders, achieving sustained virologic response rates comparable to those without psychiatric condition (96.9% *vs* 97.7%). Moreover, no specific psychiatric diagnosis was found to reduce the likelihood of treatment success (11).

The prevalence of HVC is notably higher among individuals with serious mental disorder than in the general population, which is often attributed to high-risk behaviors associated with mental health issues. This study evaluates the impact of such behaviors on blood-borne virus infections in psychiatric populations in Poland.

By the end of 2022, Poland had 61 psychiatric hospitals with 16,700 beds, serving 179,600 inpatients annually (17). Our study included over 12,000 patients, which means that it covered over 7% of psychiatrically hospitalized patients. This makes the study representative for all psychiatric patients in Poland treated in hospitals.

Recent data from Poland indicate estimate anti-HCV antibodies prevalence in the general population at ~ 0.6%, and active HCV infection (HCV RNA) at ~0.4%% (18, 19). In our study, 1.8% of psychiatric patients tested positive for anti-HCV antibodies, and 1.3% for active HCV—approximately three times higher than in the general population.

Global studies show regional variations in HCV prevalence among psychiatric populations. A 2016 review reported HCV prevalence rates of 4.9% (95% confidence interval [CI]: 3.0–7.9%) in Europe, 17.4% (95% CI: 13.2–22.6) in North America, and 3.1% (95% CI: 1.0–9.3) in Oceania (3). Given the considerable time that has elapsed since the publication of this review, we have also examined some recent studies on the prevalence of HCV in patients with mental disorders.

A study published in 2021 revealed a markedly elevated prevalence of HCV infection among psychiatric inpatients in Switzerland. The prevalence of HCV (defined as positive for anti-HCV antibodies or HCV RNA) was 10.8% in all verified admissions (total number of screened = 1681), with prevalence reaching 25.7% among those with substance use disorders *vs*. 3.5% in those without (6).

Two Spanish studies further support the link between HCV and substance use. Among the 345 analyzed patients in Salamanca, the overall prevalence of HCV was found to be 3.8%, rising to 14.3 among patients with dual diagnoses of psychiatric and substance use disorders (20) In the newest study from Spain among 404 hospitalized patients with severe mental disorders, screening identified 12 patients (3%) tested positive for anti-HCV, with 8 of these having a history of drug use (21).

In our study, active HCV prevalence was 2.7% among those with substance use disorders and 0.7% among those without—a similar risk pattern, albeit with lower overall figures.

Two recent Australian studies reported a prevalence of HCV antibodies among patients with mental disorders of 4.6% in 4492 consecutive individuals admitted to a tertiary hospital mental health service (22,) and 10.8% in 260 patients admitted to inpatient psychiatric units (23). These figures are also substantially higher than those reported in our analysis. In both Australian studies, however, key HCV predictors included intravenous drug use, incarceration, and age—paralleling our findings, except that age was not significant in our data.

A three-year French study found 2.16% anti-HCV and 0.59% active HCV prevalence among psychiatric inpatients (number of screened =2541) (24). Interestingly, the study found that among patients with HCV antibodies those with active HCV infection were statistically less likely to have a history of illicit drug use (other than cannabis) and less likely to have been previously incarcerated, with odds ratios of 0.18 and 0.23 respectively. The authors conclude that patients with mental disorders who have not yet been treated for HCV are more likely to be those without an identified risk factor for HCV, which suggests that suggesting systematic screening of patients with mental disorders is necessary even in the absence of known risk factors. Another French study found 4.2% anti-HCV and 2.2% HCV RNA positivity among 407 patients admitted to a psychiatric hospital, with drug use as the primary risk factor (25).

Overall, these international studies consistently show elevated HCV prevalence in psychiatric populations compared to general population, which is often associated with high-risk behaviors including drug use and previous incarceration.

The reported prevalence of anti-HCV antibodies in the Polish population of patients with psychiatric disorders, namely 1.8%, is lower than in the other countries. This discrepancy may be due to differences in screening practices and population characteristics. However, it should be noted that the prevalence of active infection in the Polish population of patients with psychiatric disorders is consistent with the results of the 4 newly published studies from Spain and France (reported values range from 0.5% to possibly 3.8%), despite the fact that in all these 4 studies the proportion of psychiatric patients with anti-HCV antibodies was numerically higher than in the Polish sample (2.16% to 3.8% in Spain and France compared to 1.8% in Poland). It is also noteworthy that the Polish study was the largest of all those described (including the primary studies included in the systematic review (3)).

The incorporation of the findings from the univariate statistical analysis serves to further enhance the conclusions of our study. The analysis revealed significant differences between the groups, with the highest disparities noted in past intravenous drug use, liver disease, history of cancer, blood transfusions prior to 1992 and substance misuse disorder.

These findings underscore the imperative for the implementation of targeted screening and intervention programs. In particular, the robust link between intravenous drug use and elevated HCV prevalence indicates that interventions must prioritize the development and implementation of comprehensive substance use treatment programs, in conjunction with regular HCV screening and educational initiatives. Furthermore, the high correlation between liver disease and previous HCV treatment indicates that there is a subset of the population that may benefit from ongoing healthcare monitoring to effectively manage and mitigate long-term health consequences.

The study’s findings highlight the imperative for comprehensive strategies that integrate enhanced screening measures with prompt treatment options, underscoring the efficacy of DAAs (26). This method has been demonstrated to be more efficacious in reducing HCV-related mortality than traditional infection control and harm reduction strategies currently employed for individuals who inject drugs. A Proactive, systematic, integrated, and destigmatized approach is crucial to meet the WHO’s goal of eliminating HCV as a public threat by 2030 (10).

## 5 Conclusion

In our research, anti-HCV antibodies and HCV RNA were identified in 1.8% and 1.3% of psychiatric patients respectively - about three times the rate in Poland’s general population (18, 19). These results support the need for systematic, integrated, and destigmatized interventions aligned with the WHO’s HCV elimination targets for 2030 (10).

## Data Availability

The raw data supporting the conclusions of this article will be made available by the authors, without undue reservation.
